# Novel protein acylations in ferroptosis: decoding the regulatory roles of lactylation, crotonylation, succinylation, and β-hydroxybutyrylation

**DOI:** 10.3389/fphar.2025.1688548

**Published:** 2025-12-17

**Authors:** Meiquan Wei, Shiming Wei, Xuhua Xie, Taotao Liu, Jun Deng

**Affiliations:** 1 Department of Pharmacy, The First Affiliated Hospital of Guangxi Medical University, Nanning, China; 2 The First Clinical Hospital, The First Affiliated Hospital of Guangxi Medical University, Nanning, China

**Keywords:** ferroptosis, novel protein modifications, Lactylation, crotonylation, succinylation, β-hydroxybutyrylation

## Abstract

Ferroptosis is a unique form of iron-dependent programmed cell death, characterized by the abnormal accumulation of lipid peroxides, which plays a important role in various physiological and pathological processes. Crucially, the activity and stability of core ferroptosis regulators (including GPX4, ACSL4, FSP1, and iron/lipid metabolism proteins) are dynamically controlled by diverse post-translational modifications (PTMs), positioning PTMs as central molecular switches modulating cellular ferroptotic susceptibility across pathophysiological contexts. Elucidating the regulatory mechanisms of PTMs in ferroptosis is of great significance for understanding the complex network of cell death and providing new perspectives for disease treatment and diagnosis. This review focuses on four emerging PTMs—lactylation, crotonylation, succinylation, and β-hydroxybutyrylation—and their roles in promoting ferroptosis progression via protein regulatory systems. Additionally, it explores their clinical potential as therapeutic targets and diagnostic biomarkers. The definitions, functional mechanisms, and enzymatic regulation of these PTMs are systematically elucidated.

## 1 Introduction

Cell death can be categorized into two distinct classes based on the rapidity of onset and the amenability of the process to pharmacological or genetic intervention: accidental cell death and regulated cell death (RCD) (Newton et al., 2024). Accidental cell death arises from stochastic biological events, whereas RCD is governed by defined signaling cascades and molecular mechanisms. Ferroptosis, a subset of RCD, is characterized by iron-dependent lipid peroxidation (Stockwell et al., 2017). Emerging evidence implicates ferroptosis in the pathogenesis of neurodegenerative disorders (Jeong et al., 2009; Oakley et al., 2007), ischemia-reperfusion injury (Cai et al., 2023), and oncogenic suppression, particularly in metabolically vulnerable cancer cells exhibiting heightened sensitivity to this death modality (Jiang et al., 2021). Advances in proteomic technologies have accelerated the elucidation of ferroptosis-regulatory networks, identifying critical proteins involved in iron homeostasis, lipid metabolism, and redox control (Wang and Wang, 2023; Liu X. et al., 2024). Mounting data further underscore the pivotal role of post-translational modifications (PTMs) in modulating ferroptosis susceptibility across diverse pathophysiological contexts. Exploring this intricate PTM-ferroptosis interplay holds immense therapeutic potential, offering novel avenues for targeted interventions and diagnostic biomarker discovery (Wang Y. et al., 2024).

This review seeks to comprehensively dissect the reciprocal regulatory interplay between PTMs and ferroptosis, and to evaluate their translational therapeutic potential. Initially, we will characterize the molecular underpinnings and clinical relevance of ferroptosis, assessing its viability as a therapeutic target. Subsequently, we will interrogate four emerging PTMs—lysine lactylation (Kla), crotonylation (Kcr), succinylation (Ksucc), and β-hydroxybutyrylation (Khbh)—delineating their dynamic regulatory circuits and epigenetic modulation of core ferroptosis effectors (GPX4, ACSL4, FSP1). Finally, leveraging recent breakthroughs in PTM-targeted ferroptosis modulation, we will explore innovative therapeutic paradigms that harness PTM manipulation to control ferroptosis, offering mechanistic insights and preclinical frameworks for precision medicine and organoprotection strategies.

## 2 Overview of ferroptosis

Since the term “ferroptosis” was first introduced by Brent R. Stockwell’s team in 2012 (Zhou et al., 2024), it has garnered significant attention over the past decade across disciplines including oncology, neurodegenerative diseases, and ischemia-reperfusion injury research. Ferroptosis represents an iron-dependent, non-apoptotic modality of regulated cell death, characterized by the aberrant accumulation of intracellular lipid peroxides that disrupts cellular membrane integrity (Dixon et al., 2012). Its defining features and mechanistic underpinnings can be categorized into three primary aspects, outlined below.

### 2.1 Abnormal iron metabolism

The accumulation of iron ions represents a critical determinant in ferroptosis (Ru et al., 2024). Iron ions are sourced from two primary pathways: ferritinophagy, the autophagic degradation of ferritin (Wang Y. et al., 2024; Dai et al., 2024), and transferrin receptor 1 (TfR1)-mediated iron uptake. Specifically, circulating iron ions in the bloodstream bind to transferrin, which facilitates their endocytosis into cells via TfR1. Following internalization, iron ions are reduced from Fe^3+^ to Fe^2+^ and released into the labile iron pool (LIP)—a dynamic intracellular reservoir of loosely bound, redox-active iron. Within the LIP, unstable Fe^2+^ ions catalyze Fenton reactions, generating hydroxyl radicals that trigger the accumulation of lipid peroxides. This cascade ultimately culminates in oxidative cell death (Ru et al., 2024; Berndt et al., 2024). In conclusion, the close relationship between iron and ferroptosis deserves in - depth investigation, especially in terms of maintaining normal physiological activities and ensuring proper organ function in the body (Zhang, 2024).

### 2.2 Accumulation of lipid peroxides

The accumulation of lipid peroxides represents the central mechanism underlying ferroptosis (Liang et al., 2022). During this process, polyunsaturated fatty acids (PUFAs), notably arachidonic acid (AA) and adrenic acid (AdA), are particularly prone to oxidative damage (Zhang, 2024). Under the catalytic activity of long-chain fatty acid-CoA ligase 4 (ACSL4) and lysophosphatidylcholine acyltransferase 3 (LPCAT3), PUFAs are esterified into phospholipid-polyunsaturated fatty acids (PL-PUFAs). These PL-PUFAs subsequently undergo peroxidation via ALOX-mediated reactions, which amplify lipid peroxidation cascades and drive ferroptotic cell death (Jiang et al., 2021).

### 2.3 Imbalance of redox homeostasis

**FIGURE 1:**
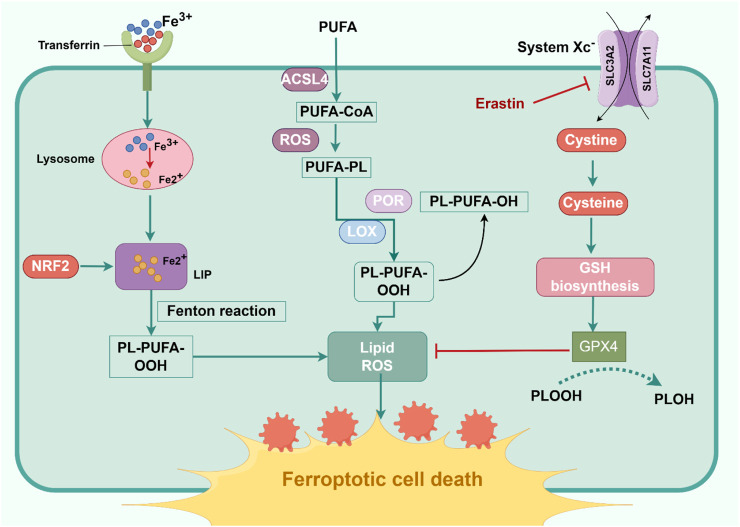
Mechanism and Process of Ferroptosis. Ferroptosis occurs in three key stages: disruption of iron metabolism, lipid peroxidation, and imbalance of the antioxidant system. Cells uptake iron ions from the blood through transferrin receptor 1 (TFR1), where they are reduced to ferrous ions (Fe^2+^) and stored in an unstable iron pool (LIP). These ferrous ions are highly reactive and generate hydroxyl radicals via the Fenton reaction. These radicals attack polyunsaturated fatty acids in the cell membrane, initiating lipid peroxidation. Polyunsaturated fatty acids, such as arachidonic acid, are oxidized by the combined action of iron ions and oxidases, leading to the formation of lipid peroxides. These peroxides disrupt the integrity of the cell membrane, ultimately causing cell death. Glutathione peroxidase 4 (GPX4) is central to the antioxidant defense, reducing lipid peroxides to non-toxic compounds with the help of glutathione (GSH). Concurrently, FSP1 neutralizes lipid radicals by reducing CoQ10, working in tandem with GPX4 to form a dual defense system. However, when iron accumulation becomes excessive or GPX4 activity is compromised, the antioxidant system is disrupted, leaving the cell unable to counter lipid peroxidation, resulting in ferroptosis.

During normal cellular metabolism, cells produce both substrates and oxidants that promote lipid peroxidation, as well as inhibitory molecules that counteract this process (Pope and Dixon, 2023). Ferroptosis is intimately associated with lipid peroxidation, occurring when endogenous defense systems fail to prevent the accumulation of peroxidized lipids (Jiang et al., 2021). Central to these protective mechanisms is the glutathione-dependent antioxidant system, wherein glutathione peroxidase 4 (GPX4) serves as the principal regulator of ferroptosis (Liang et al., 2022). GPX4 uniquely catalyzes the reduction of membrane lipid hydroperoxides using glutathione (GSH), thereby mitigating peroxidative damage and maintaining cellular homeostasis (Alves et al., 2025). By converting peroxidized phospholipids (PUFA-PL-OOHs) into inert alcohol derivatives (PUFA-PL-OHs), GPX4 neutralizes ferroptotic stimuli (Liu J. et al., 2024). Beyond GPX4-dependent pathways, ferroptosis resistance is mediated by alternative mechanisms, including ferroptosis suppressor protein 1 (FSP1), dihydroorotate dehydrogenase (DHODH), and GTP cyclohydrolase 1 (GCH1)-tetrahydrobiopterin (BH4) pathways (Alves et al., 2025). FSP1 reduces ubiquinone to ubiquinol, attenuating lipid radical propagation and promoting vitamin E-mediated antioxidant defense (Doll et al., 2019; Yang J-S. et al., 2025). DHODH produces mitochondrial coenzyme Q hydroquinone (CoQH_2_), which inhibits lipid peroxidation (Mao et al., 2021). GCH1 synthesizes BH4, a lipophilic antioxidant that remodels membrane lipid composition and enhances CoQ10 biosynthesis, while simultaneously reducing pro-ferroptotic PUFA-PL levels (Kraft et al., 2020). Emerging evidence further implicates metabolic reprogramming, AMPK signaling, squalene accumulation, and other factors in ferroptosis regulation, underscoring the complexity of this regulated cell death modality (Figure 1).

## 3 Overview of novel protein modifications

Before detailing the four emerging protein post-translational modifications (PTMs) central to this review, it is essential to summarize the well-established “classical PTMs” in ferroptosis regulation, as they provide a foundational framework for understanding the functional roles of novel modifications. Among these classical PTMs, acetylation is the most extensively characterized. Histone acetylation (e.g., H3K27ac) activates the transcription of anti-ferroptosis genes (e.g., GPX4 and FSP1) by relaxing chromatin structure. Conversely, histone deacetylase inhibitors (e.g., TSA) upregulate GPX4 expression by increasing global acetylation levels, thereby suppressing ferroptosis (Berndt et al., 2024; Oliveira et al., 2021). In non-histone proteins, acetylation (e.g., at ACSL4 lysine 383) promotes proteasomal degradation, attenuating lipid peroxidation-mediated ferroptosis (Zhou P. et al., 2025). In the case of methylation, histone mark H3K4me3 enhances the transcription of iron metabolism-related genes (e.g., TfR1) by binding to their promoter regions, promoting iron accumulation (Wang et al., 2025a). In contrast, DNA methylation silences SLC7A11 expression, a key subunit of system Xc^−^, thereby reducing glutathione synthesis and increasing cellular susceptibility to ferroptosis (Lohoff et al., 2022). Phosphorylation, another core classical modification, directly regulates the activity of pivotal ferroptosis proteins. For example, phosphorylation of ACSL4 at Ser278 enhances its enzymatic capacity to esterify polyunsaturated fatty acids into phospholipids (PL-PUFAs), accelerating lipid peroxidation (Berndt et al., 2024; Lin et al., 2025). In opposition, phosphorylation of GPX4 S104 inhibits its antioxidant activity, indirectly promoting ferroptosis (Wu et al., 2023).

In contrast to classical modifications—which are predominantly enzyme-catalyzed and operate through relatively well-defined pathways—the novel modifications emphasized in this review (lactylation, crotonylation, succinylation, and β-hydroxybutyrylation) are distinguished by their direct mediation by metabolites (e.g., lactate, succinyl-CoA, β-hydroxybutyrate). This characteristic enables a tighter coupling between cellular metabolic states and ferroptosis regulation, thereby providing new perspectives for deciphering ferroptosis mechanisms in pathological microenvironments such as high-lactate tumors and glucose-deficient ischemic tissues.

### 3.1 Overview of lactylation modification

The core function of protein lactylation is to covalently modify proteins, including histones and non-histones, through lactate, thereby dynamically linking the cellular metabolic state to functional regulation. The mechanism entails lactate directly acting as a metabolic signaling molecule, modifying lysine residues via ester bonds. This modification alters the structure or activity of target proteins. For instance, the addition of lactate groups to histone lysine residues neutralizes the positive charge of lysine and changes the interaction between histones and DNA, leading to chromatin decondensation. This process promotes gene transcription and influences the onset and progression of various diseases. Non-histone lactylation modifications can also impact the development of various diseases by altering protein structures and functions (Chen et al., 2025). Lactylation regulates the activity of metabolic enzymes and modulates intracellular metabolic pathways. Moreover, it is implicated in diverse pathological conditions—including tumors, cardiovascular diseases, and neurodegenerative disorders—and alterations in its levels may serve as potential biomarkers for disease diagnosis and treatment monitoring (Xu K. et al., 2024; Zhang et al., 2025).

Similar to other post-translational modifications (PTMs), lactylation involves enzymatic mechanisms such as “writers,” “erasers,” and “readers.” The rapid advancement in the study of lactylation modifications has revealed an increasing number of lactylation writers, such as TIP60 (Chen H. et al., 2024), KAT8 (Xie et al., 2024), and AARS1 (Zong et al., 2024). Regarding de-lactylation modifications, similar to other histone post-translational modifications (HPTMs), HDAC3 and SIRT1-3 also possess de-lactylation capabilities. The difference lies in the fact that HDAC3 acts as an NBS1 de-lactylase (Chen H. et al., 2024), while SIRT3 deviates from the classic role of deacetylases by inhibiting the proliferation of hepatocellular carcinoma by targeting the lactylation modification of the Cyclin E2 protein (Jin et al., 2023). In the “reader” mechanism, research on lactylation readers is still limited. Hu et al. found that Brg1 (SMARCA4) specifically recognizes the H3K14la modification through its bromodomain and subsequently recruits histone acetyltransferase complexes to activate the expression of metabolism-related genes (Hu et al., 2024) (Table 1).

**TABLE 1:** Writers, Erasers and Readers of lactylation.

Category	Name	Histone lactylation site	Non-histone protein	References
Writers	p300	H3K18	YY1, α-MHC, HMGB1, NCL, PKM2, Twist1, MeCP2, Snail1	Liu et al. (2025a), Li et al. (2022), Fan et al. (2023), Wang et al. (2023), Zhang et al. (2023b), Huang et al. (2024b), Chen et al. (2024c), Yang et al. (2024b), Xu et al. (2024b)
	GTPSCS	H3K18	ND	Liu et al. (2025a)
	HBO1	H3K9	ND	Niu et al. (2024)
	CBP	ND	HMGB1, MRE11, Twist1, Snail1	Fan et al. (2023), Xu et al. (2024b), Yang et al. (2022), Chen et al. (2024d)
	TIP60	ND	NBS1, Vps34	Chen et al. (2024a), Chen et al. (2024e)
	KAT8	ND	eEF1A2, LTBP1	Xie et al. (2024), Zou et al. (2024)
	AARS1	ND	p53, METTL16	Zong et al. (2024), Sun et al. (2023)
	AARS2	ND	PDHA1, CPT2, METTL16	Sun et al. (2023), Mao et al. (2024)
	ATAT1	ND	NAT10	Yan et al. (2024)
	HDAC6	ND	α-tubulin	Sun et al. (2024a)
	NAA10	NDA	NSUN2	Niu et al. (2025)
Erasers	HDAC1	H3K9, H3K18, H3K23,H4K5, H4K8, H4K12	ND	Moreno-Yruela et al. (2022)
	HDAC2	H3K9, H3K18, H3K23,H4K5, H4K8, H4K12	ND	Moreno-Yruela et al. (2022)
	HDAC3	H3K9, H3K18, H3K23,H4K5, H4K8, H4K12	NBS1, MeCP2	Chen et al. (2024a), Chen et al. (2024c), Moreno-Yruela et al. (2022)
	SIRT1	H3K18, H4K5	CNPY3, α-MHC, PTBP1	Zhang et al. (2023b), Chen et al. (2024c), Moreno-Yruela et al. (2022), Zhou et al. (2025b)
	SIRT2	H3K18, H4K5	METTL16	Sun et al. (2023), Moreno-Yruela et al. (2022)
	SIRT3	H3K18, H4K5	CCNE2, PDHA1, CPT2	Mao et al. (2024), Moreno-Yruela et al. (2022), Jin et al. (2023)
Readers	Brg1	H3K14	ND	Hu et al. (2024)
	TRIM33 Bromodomain	H3K18	ND	Nuñez et al. (2024)

### 3.2 Overview of crotonylation modification

Protein crotonylation (Kcr) is a post-translational modification marked by a conjugated double bond, distinguishing it from other protein acylation modifications as it can occur on serine residues in addition to lysine residues (Liao et al., 2020). Previous studies have conducted genomic analyses on histone crotonylation, revealing that histone Kcr influences the latency and reactivation of HIV (Zhang H. et al., 2023), and mediates the reprogramming of tumor immunity (Yuan et al., 2023). Histone Kcr enhances gene expression to a greater extent than the extensively studied lysine acetylation (Kac), suggesting that Kcr may represent a novel potential therapeutic target (Westerveld et al., 2025). Several “writers” involved in histone crotonylation modification include common acetyltransferases such as p300 (Gao et al., 2024), GCN5 (Han et al., 2024) and KAT7 (Yan et al., 2022). This metabolic regulatory network has expanded beyond the classical chromatin field to encompass new enzymatic systems: the multifunctional metabolic enzyme ACSS2 functions as the biological “writer” for H3K9cr (Li L. et al., 2024). Currently, the known de-crotonylating enzymes primarily belong to the histone deacetylase (HDAC) family and the Sirtuin family. Experiments have demonstrated that HDAC1,HDAC2 and HDAC8 can also exhibit pyruvate dehydrogenase activity *in vitro* (Li D. et al., 2024; Madsen and Olsen, 2012). Moreover, SIRT1, SIRT2, and SIRT3 have all been shown to possess histone de-crotonylase activity *in vitro* (Feldman et al., 2013; Bao et al., 2014; Liao et al., 2023). There are currently three known types of crotonylation “readers”: YEATS domain proteins, double PHD finger domain proteins, and bromodomain proteins, each with distinct functional roles. Among them, the YEATS domain is the preferred reader for crotonylated lysine. Compared to YEATS domain proteins, the other two protein families are less proficient in recognizing crotonylation modifications (Table 2).

**TABLE 2:** Writers, Erasers and Readers of crotonylation.

Category	Name	Histone crotonylation site	Non-histone protein	References
Writers	p300	H3K18	IDH1, FASN, MTHFD1	Gao et al. (2024), Zheng et al. (2023)
	CBP	H3K18	NPM1, DDX5, ENO1,IDH1, FASN, MTHFD1	Zheng et al. (2023), Yang et al. (2023), Sabari et al. (2015), Hou et al. (2021)
	GCN5* (ADA)	H3K9, H3K14, H3K18, H3K23, H3K27	DNA-PKcs (K525)	Han et al. (2024), Li et al. (2025), Kollenstart et al. (2019)
	hMOF	H3K4, H3K9, H3K18, H3K23, H4K8, H4K12	NPM1	Yang et al. (2023), Xu et al. (2017)
	Esa1(Piccolo NuA4)	H4K5,H4K8,H4K12 H4K16	ND	Kollenstart et al. (2019)
	ACSS2	H3K9	ND	Li et al. (2024a)
	HAT1*	H3K9	ND	Andrews et al. (2016)
	RTT109*	H3K9	ND	Andrews et al. (2016)
	PCAF	ND	NPM1,DDX5	Xu et al. (2017)
	HBO1	H3K14,H4K12	ND	Xiao et al. (2021)
	KAT7	ND	CANX (K525)	Yan et al. (2022)
	TIP60	ND	EB1	Song et al. (2021)
	PCAF	ND	PGK1,Ku80	Guo et al. (2024a), Lao et al. (2024)
	YjgM	ND	PmrA(Lys 164)	Zhuang et al. (2024)
Erasers	HDAC1	H3K4, H3K9, H3K23, H4K8, H4K12, H3K18	NPM1,IDH1, FASN, MTHFD1	Zheng et al. (2023), Xu et al. (2017), Qian et al. (2023), Wei et al. (2017)
	HDAC2	H3K9, H3K23, H4K8, H4K12, H3K18, H2BK12	ND	Wei et al. (2017), Yang et al. (2021b)
	HDAC3	NH3K9, H4K8, H3K18	NPM1,AKT1,IDH1, FASN, MTHFD1	Zheng et al. (2023), Xu et al. (2017), Qian et al. (2023), Wei et al. (2017)
	HDAC6	ND	Lamin A (K265/ 270)	Zhang et al. (2022)
	HDAC7	ND	14–3–3ε (K73, K78)	Zheng et al. (2022b)
	HDAC8	H3K4, H3K9, H3K23, H4K8, H4K12, H3K18	Ku80	Lao et al. (2024), Wei et al. (2017), Yang et al. (2021b)
	SIRT1	H3K9,H4K8, H3K4, H3K18, H2AK119	ND	Feldman et al. (2013), Wei et al. (2017), Hao et al. (2022)
	SIRT2	H3K9	ENO1	Feldman et al. (2013), Hou et al. (2021)
	SIRT3	H3K4,H3K27	cGAS	Bao et al. (2014), Liao et al. (2023), Guo et al. (2024b)
	SIRT6	H3K27	ND	Liao et al. (2022)
	SIRT7	ND	PHF5A (K25), RRM2(K283)	Yu et al. (2021), Sun et al. (2024b)
	FOSIR5*	H3K18	ND	Zhang et al. (2021)
Readers	TAF14	H3K9	ND	Andrews et al. (2016)
	YEATS2	H3K27	ND	Zhao et al. (2016)
	AF9	H3K9,H3K18,H3K27	ND	Li et al. (2016a)
	MOZ,DPF2,TAF1	H3K14	ND	Li et al. (2016a), Flynn et al. (2015)

### 3.3 Overview of succinylation modification

Since its initial report in 2011, lysine succinylation (Ksucc) has been established as an evolutionarily conserved and widely distributed post-translational modification. It plays a crucial role in chromatin dynamics, alongside lysine acetylation (Kac) (Zheng et al., 2023). This modification introduces a charged succinyl group (-CO-CH2-CH2-COO^-^), which significantly alters nucleosome conformation, reduces histone-DNA binding affinity, and promotes chromatin unwinding and transcriptional activation. Conversely, the de-succinylation process enhances genomic superhelical stability and inhibits gene expression (Zhang et al., 2011; Weinert et al., 2013). Succinylation constitutes a key mechanism that enables cellular adaptation to environmental changes and maintenance of homeostasis through the dynamic regulation of protein functions, thus contributing to cellular metabolism, signal transduction, and disease pathogenesis (Weinert et al., 2013).

Similar to other post-translational modifications (PTMs), succinyltransferases have been identified in the succinylation process. Examples of these enzymes include histone acetyltransferase (HAT1) (Yang G. et al., 2021) and oxoacid-CoA:3-ketoacid-CoA transferase 1 (OXCT1) (Ma et al., 2024). These enzymes utilize succinyl-CoA as a substrate to catalyze the succinylation of lysine residues, without altering the succinyl-CoA content during this process. Like acetylation and deacetylation, succinylation also has its de-succinylation counterparts. COBb was the first de-succinylase discovered in prokaryotes, and it also exhibits deacetylation activity (Colak et al., 2013). In eukaryotes, SIRT5 (Zhang M. et al., 2019) and SIRT7 (Hu et al., 2025) are the known de-succinylases. Additionally, a histone succinylation “reader” has been identified: GAS41. The YEATS domain of this protein binds significantly to H4K122succ and can recognize various types of acylation modifications under different pH conditions (Wang Y. et al., 2018) (Table 3).

**TABLE 3:** Writers, Erasers and Readers of succinylation.

Category	Name	Histone succinylation site	Non-histone protein	References
Writers	KAT2A (GCN5)	H3K79	ND	Wang et al. (2017)
	OXCT1	ND	LACTB (K284)	Ma et al. (2024)
	HAT1	H3K122	PGAM1	Yang et al. (2021a)
	CPT1A	ND	Lysine (K80,K81 K335)	Kurmi et al. (2018)
Erasers	HDAC1	H3K14, H3K23, H3K18,H3K37 H4K5,H4K16 H4K20	ND	Li et al. (2023)
	HDAC2	H3K14, H3K23	ND	Li et al. (2023)
	HDAC3	H3K14, H3K23	ND	Li et al. (2023)
	SIRT5	ND	CPS1,Atp5b, IDH2, SOD1, ACAD9	Zhang et al. (2019a), Wang et al. (2018b), Rardin et al. (2013)
	SIRT7	H3K122	ND	Li et al. (2016b)
	CobB	ND	Lysine	Colak et al. (2013)
Readers	GAS41	H3K122	ND	Wang et al. (2018a)

### 3.4 Overview of β-hydroxybutyrylation modification

β-Hydroxybutyrate (BHB) is a crucial ketone body, and its levels increase under low-carbohydrate conditions, such as fasting, intermittent fasting, or ketogenic diets. When the glucose supply to the body is limited, BHB can serve as the primary energy source for metabolically active tissues, including the brain. Studies have demonstrated that BHB can bind to free coenzyme A (CoA) to form BHB-CoA, and this conjugate can undergo β-hydroxybutyrylation modifications (Kbhb) at lysine residues (Moya-Garzon et al., 2025; Qin et al., 2024). Through the regulation of gene expression, metabolic pathways, and cellular functions, β-hydroxybutyrate (BHB) plays a critical role in energy metabolism, disease development, and therapeutic development. This positions BHB as a key mechanism connecting metabolic signaling with epigenetic control (Newman and Verdin, 2017).

Similar to other acetylation modifications, Kbhb modifications are also regulated by enzymatic reactions. It has been found that the acetyltransferase P300 can catalyze the modification of histone Kbhb levels at H3K9, H3K18, H3K27, and H4K8 sites, and some of these Kbhb modifications are more sensitive to p300 regulation than their corresponding Kac sites (Huang et al., 2021). The “erasers” of Kbhb are primarily HDAC1, HDAC2, and SIRT3. They mediate the removal of Kbhb through their enzymatic activities. However, the activity of SIRT3 on Kbhb depends on the modified sites. For instance, SIRT3 preferentially removes Kbhb modifications at specific histone sites, such as H3K9bhb, which differs from its broad site-specificity toward Kcr modifications (Zhang X. et al., 2019). As the first identified “reader” of β-hydroxybutyrylation, ENL recognizes H3K9bhb at gene promoters through its YEATS domain and recruits other transcription factors to chromatin to regulate the gene transcription process (Chen C. et al., 2024) (Table 4).

**TABLE 4:** Writers, Erasers and Readers of β-hydroxybutyrylation.

Category	Name	Histoneβ-hydroxybutyrylation site	Non-histone protein	References
Writers	P300	H3K9,H3K18, H4K8	ND	Huang et al. (2021)
	CBP	H3K18, H4K8	ND	Huang et al. (2021)
Erasers	HDAC1	H3K9,H3K18 H4K9	ND	Huang et al. (2021)
	HDAC2	H3K9 H3K18 H4K9	ND	Huang et al. (2021)
	SIRT3	H3K4,H3K9, H3K14, H3K18, H3K23, H3K27, H4K16	ND	Zhang et al. (2019b)
Readers	ENL	H3K9	ND	Chen et al. (2024b)

## 4 Novel protein modifications in ferroptosis

Post-translational modifications (PTMs) denote the covalent addition of diverse chemical functional groups to specific amino acid residues of proteins following translation, thereby modifying their structural and functional characteristics (Shang et al., 2022). This section primarily elucidates the definitions, functions, and enzymatic mechanisms of lactylation, crotonylation, succinylation, and β-hydroxybutyrylation modifications, focusing on four out of the nine previously delineated PTMs (Table 5).

**TABLE 5:** The relationship between lactylation, crotonylation, succinylation, β-hydroxybutyrylation, and ferroptosis-related proteins.

PTM category	Substrate	Site	Classification	Downstream	Disease	References
Lactylation	H3K18	ND	Histone protein	METTL3,ACSL4	Acute lung injury	Wu et al. (2024)
	H3K14	ND	histone protein	TFRC,SLC40A1	Acute lung injury	Gong et al. (2025)
	HDAC1	K412	Histone deacetylase	H3K27ac,FSP1 m6A	Colorectal Cancer	Yang et al. (2025b)
	NSUN2	K508	Methyltransferase	GCLC m5C	Gastric cancer	Niu et al. (2025)
	PCK2	K100	Carboxykinase	OXSM	Liver transplantation IRI	Yuan et al. (2025)
	METTL3	ND	Methyltransferase	TFRC m6A	Intracerebral hemorrhage	Zhang et al. (2023c)
	MDH2	K241	TCA cycle enzyme	ATP,ROS,ACLS4,GPX4	Ischemic heart disease	She et al. (2024)
	TAU	K677	Neurofilament protein	SLC7A11,Nrf2,HO-1,Bcl-2,GPX4	Alzheimer’s disease	An et al. (2024)
Crotonylation	H3K27	ND	histone protein	SQSTM1,ACSL4	Diabetic	Li et al. (2025)
	MTHFD1	Lys354,Lys553	Metabolic enzymes	NRF2,SLC7A11,GCLC,GSS	Pancreatic cancer	Zheng et al. (2023)
Succinylation	GLS	K311	Kidney-type glutaminase	Glutamate,GSH	Pancreatic ductal adenocarcinoma	Tong et al. (2021)
	ACSL4	K661	Metabolic enzymes	ROS	Ischemia–Reperfusion Injury	Liu et al. (2025b)
	HOXA5	ND	Homeobox A5 transcription factor	FSP1	Sepsis induced-lung injury	Wang et al. (2025b)
β-hydroxybutyrylation	H3K9	ND	histone protein	Gpx4, *C*th, Gclc, Acsl3, Chmp5, Gch1, Hspb1, Pcbp2	acute liver failure	Zheng et al. (2022a)

### 4.1 Lactylation

**FIGURE 2:**
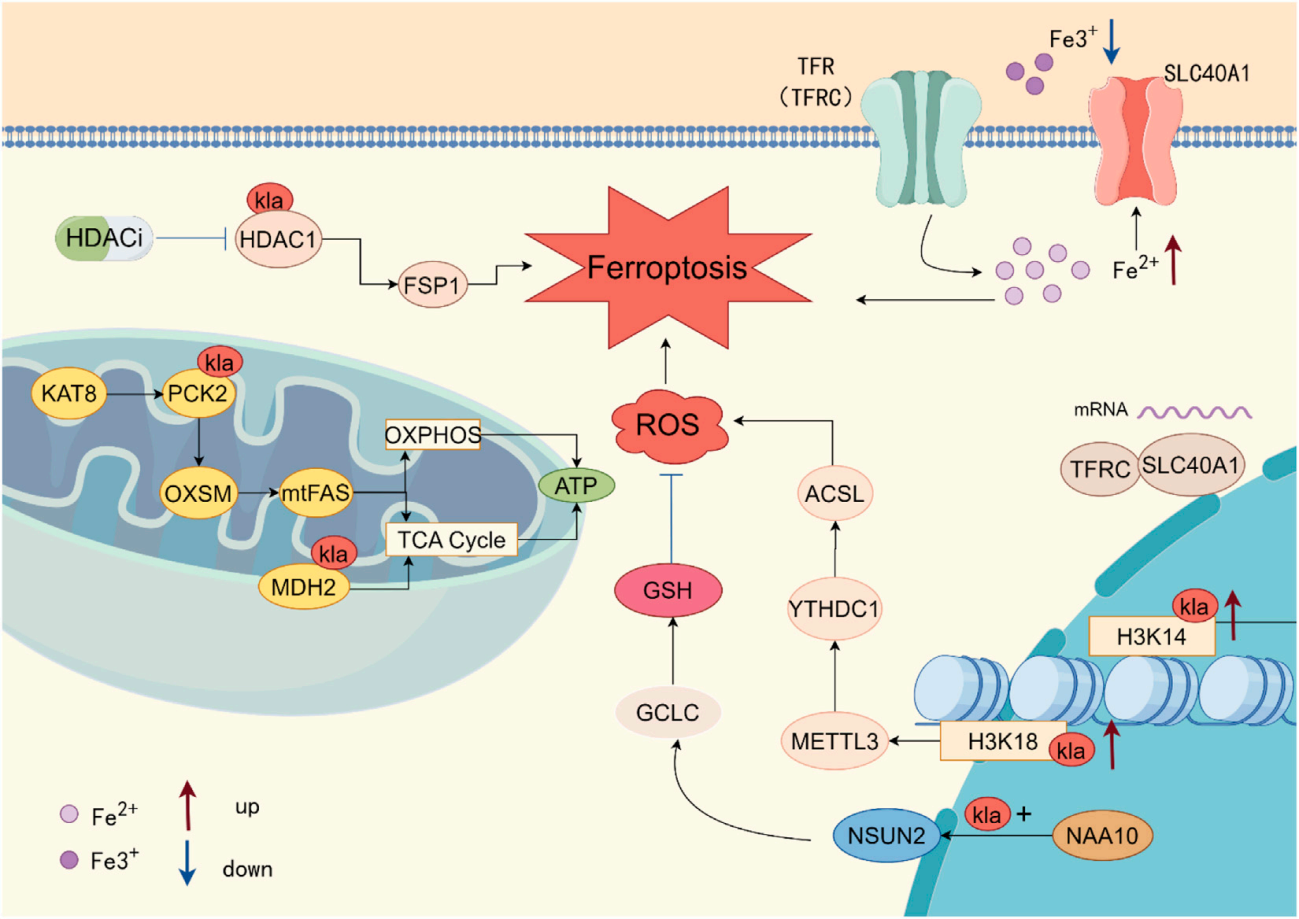
Lactylation in Ferroptosis. Direct lactylation of H3K14, H3K18, NSUN2, MDH2, PCK2, and HDAC1 by various kinases is involved in the regulation of ferroptosis. Kla, Lactylation.

The relationship between lactylation and ferroptosis has emerged as a significant research area in recent years. These two processes demonstrate potential cross-talk in metabolic reprogramming, oxidative stress, and disease regulation, particularly in the context of tumors. This section introduces protein lactylation (Kla) modifications in ferroptosis at both the histone and non-histone levels (Figure 2).

#### 4.1.1 Lactylation of the histone pathway

Emerging evidence indicates that lactylation of various proteins, including histones (H3K14, H3K18), microtubule-binding proteins (Tau), malate dehydrogenase 2 (MDH2), and RNA methyltransferases (NSUN2), plays a role in the regulation of ferroptosis.

Studies have demonstrated that histone lactylation is an epigenetic modification influenced by intracellular lactate levels, and it significantly contributes to ferroptosis by promoting gene transcription within the chromatin (Chen et al., 2025). In a high lactate microenvironment, H3K14la accumulates in the promoter regions of ferroptosis-related genes, such as transferrin receptor (TFRC) and solute carrier family 40 member 1 (SLC40A1), leading to chromatin opening and transcriptional activation. This process disrupts intracellular iron homeostasis, specifically by inhibiting iron efflux mediated by SLC40A1 and enhancing transferrin-dependent iron uptake, which triggers iron overload and lipid peroxidation cascades, ultimately driving endothelial cell ferroptosis (Gong et al., 2025).

Further research has shown that lactate exerts a dual regulatory effect on ferroptosis through histone lactylation (H3K18la): in specific pathological environments, on one hand, H3K18la activates NSF1 transcription, promoting its role as a vesicle fusion protein that stabilizes SLC7A11 (the System Xc^−^ subunit), enhancing cysteine uptake and maintaining GSH synthesis, thus inhibiting lipid peroxidation and mediating ferroptosis resistance. On the other hand, lactate mediates the H3K18la modification through p300 binding to the METTL3 promoter region, upregulating its expression. METTL3 then enhances the stability of ACSL4 mRNA via m6A methylation, promoting the esterification of polyunsaturated fatty acids (PUFAs) into acyl-CoA, leading to lipid peroxidation accumulation and exacerbating ferroptosis. These two pathways reveal the bidirectional role of the lactate-H3K18la axis in ferroptosis regulation: maintaining antioxidant defense via NSF1-SLC7A11 or driving lipid peroxidation through METTL3-ACSL4. The final effect may depend on the cell type, microenvironment, or disease stage (Huang et al., 2025; Wu et al., 2024).

Notably, in contrast to studies that activate gene transcription via H3K18la, in endothelial cells, LPS-induced H3K14la activates TFRC transcription but inhibits SLC40A1 transcription. Whether there are potential transcription factors responsible for H3K14la regulation of iron homeostasis-related genes remains to be further explored.

#### 4.1.2 Lactylation of the non-histone protein pathway

Tau, a microtubule-associated protein predominantly found in neurons, is implicated in the pathogenesis of neurodegenerative diseases, including Alzheimer’s disease. When lactylation modification occurs, it plays a distinct role in the regulation of ferroptosis. Specifically, the lactylation level of Tau at the K677 site significantly increases, activating ferritin autophagy, which releases free iron ions (Fe^2+^) and exacerbates ferroptosis (An et al., 2024). Conversely, lactylation of malate dehydrogenase 2 (MDH2) results in a substantial increase in its levels, leading to mitochondrial metabolic collapse and a burst of reactive oxygen species (ROS), ultimately triggering ferroptosis (She et al., 2024). In contrast, lactylation mediated by the lactyltransferase NAA10 at NSUN2 (at sites K216, K389) causes a decrease in GSH levels and GPX4 activity, thereby inducing resistance to ferroptosis in gastric cancer cells (Niu et al., 2025). Interestingly, as a histone deacetylase, HDAC1 primarily reduces histone acetylation and suppresses gene transcription. However, recent studies have demonstrated that histone deacetylase inhibitors (HDACi), by specifically targeting HDAC1, reduce lactylation at the K412 site, ultimately enhancing the sensitivity of colorectal cancer (CRC) to ferroptosis (Yang Z. et al., 2025).

These groundbreaking findings not only elucidate the molecular logic of non-histone lactylation modifications in ferroptosis regulation but also offer a new perspective for the development of cross-disease precision therapeutic strategies.

### 4.2 Crotonylation

Histone lysine crotonylation, an important epigenetic modification, is extensively involved in key biological functions such as gene transcription regulation, DNA damage repair, and cell cycle progression (Yang et al., 2023).

Recent research has demonstrated that crotonylation modification exhibits a complex, disease-specific dual mechanism in the regulation of ferroptosis. In high-glucose microenvironments, keratinocytes significantly upregulate intracellular crotonyl-CoA levels through metabolic reprogramming, specifically activating the catalytic activity of histone acetyltransferases like p300. This leads to crotonylation at the H3K18 and H3K27 sites. This epigenetic mark relaxes the chromatin structure, directly binds to the promoter region of the ACSL4 gene, and activates its transcription, thereby driving this core ferroptosis regulatory molecule to catalyze the esterification of polyunsaturated fatty acids (PUFAs) into membrane phospholipids. Ultimately, the accumulation of lipid peroxidation triggers keratinocyte ferroptosis (Li et al., 2025).

Notably, Liu et al. (2017) found in studies on pancreatic ductal adenocarcinoma (PDAC) that the crotonylation modification of the metabolic enzyme MTHFD1 exhibits an opposite function: its low crotonylation state enhances one-carbon metabolism activity, increasing NADPH production and inhibiting oxidative stress, thus suppressing ferroptosis in tumor cells and promoting malignant progression (Zheng et al., 2024). This switch in modification targets reveals the functional heterogeneity of crotonylation on both histones and non-histones. In addition, multidimensional studies on acute kidney injury (Li Y. et al., 2024), depression (Liu et al., 2017), and hypertrophic cardiomyopathy (Li et al., 2025) further support its broad pathological regulation.

**FIGURE 3:**
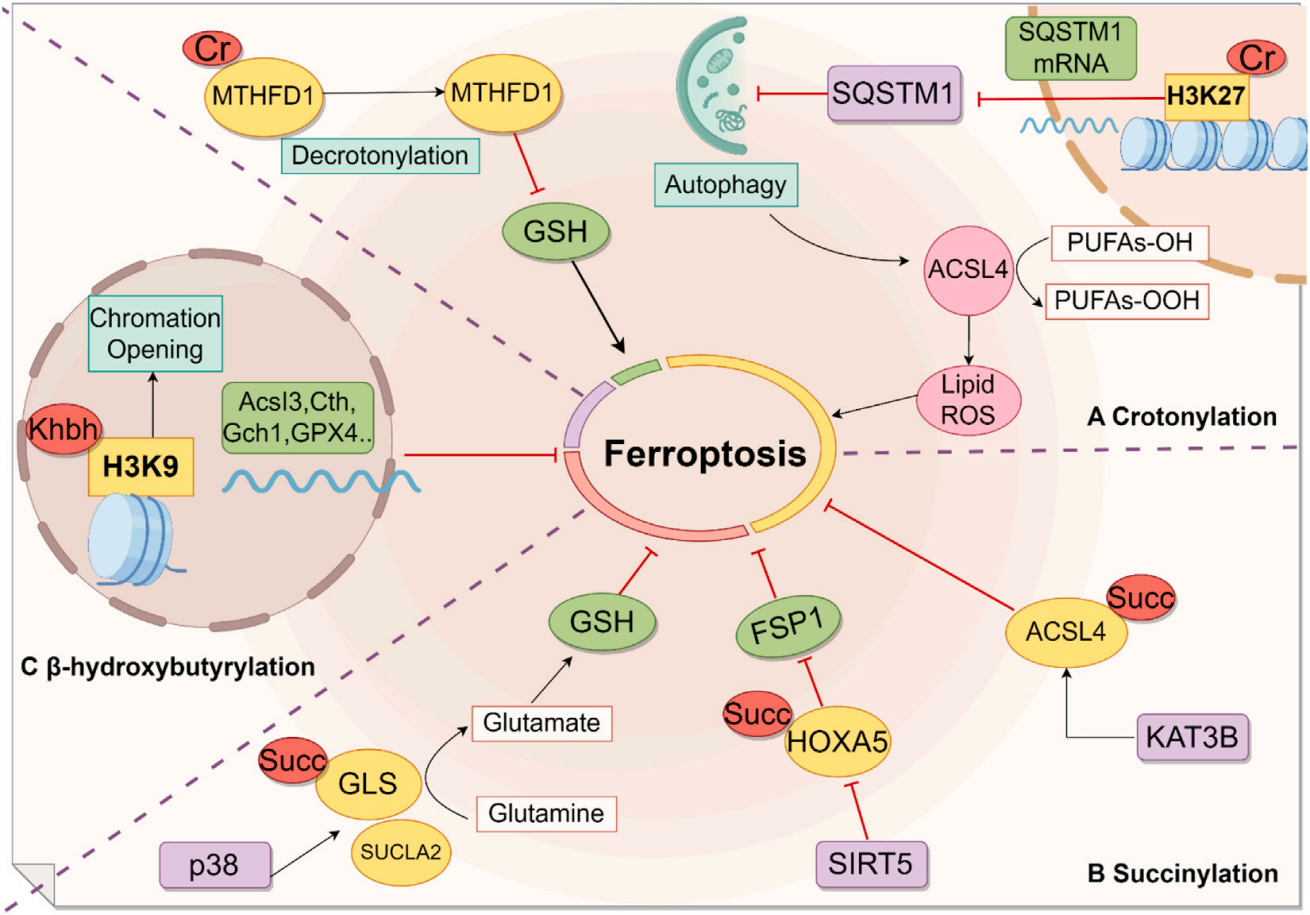
Modifications in Ferroptosis by Crotonylation, Succinylation, and β-Hydroxybutyrylation. A. Crotonylation modifies key regulatory factors in ferroptosis, such as H3K27 and MTHFD1.B. Succinylation serves as a modification mechanism for a range of proteins, including ACSL4, HOXA5, and GLS, thereby influencing the ferroptosis process.C. H3K9 plays a pivotal role in ferroptosis regulation through β-hydroxybutyrylation. Cr, crotonylation. Succ, succinylation. Khbh, β-hydroxybutyrylation.

However, as a novel post-translational modification, the core regulatory components of crotonylation—its “writers” (acyltransferases), “erasers” (deacylases), and “readers” (effector proteins)—have yet to be fully identified. This limits the precise understanding of its mechanisms in ferroptosis. Future in-depth analysis of the crotonylation toolkit will provide important target reserves and mechanistic support for the treatment of ferroptosis-related diseases (Figure 3).

### 4.3 Succinylation

Recent studies have demonstrated that the succinylation modification network is closely linked to the regulation of ferroptosis; however, its molecular mechanisms, particularly the role of histone succinylation, remain to be elucidated. Wang et al. elucidated the key role of the succinate metabolism-ferroptosis axis in a brain ischemia model: ischemic stress activates succinate dehydrogenase (SDH), resulting in the abnormal accumulation of succinate. This, in turn, generates reactive oxygen species (ROS) through reverse electron transport (RET) at mitochondrial complex I, directly triggering neuronal ferroptosis (Wang X. et al., 2024). This finding suggests that an imbalance in succinate metabolic homeostasis may serve as an important inducer of ferroptosis.

In the realm of non-histone succinylation, research on the tumor microenvironment provides new insights for mechanism analysis. Tong et al. confirmed that under nutrient stress conditions, succinyl-CoA ligase SUCLA2 forms a protein complex with glutaminase GLS, catalyzing the succinylation modification of lysine 158 in GLS (K158succ). This modification allosterically activates GLS enzymatic activity, promoting the conversion of glutamine into glutamate and enhancing the biosynthesis of glutathione (GSH). Ultimately, this aids tumor cells in establishing an ROS clearance system and resisting ferroptosis (Tong et al., 2021).

These studies not only reveal the bidirectional role of succinylation modification in regulating ferroptosis through metabolic reprogramming—both mediating pro-death signals (such as the SDH-succinate-ROS axis) and enhancing antioxidant defense (such as the SUCLA2-GLS-GSH axis)—but also imply that histone succinylation may be involved in the expression of ferroptosis-related genes through epigenetic regulation. Further analysis of the specificity of succinylation modification sites, their spatiotemporal dynamics, and their interactions with ferroptosis-related signaling pathways will offer new strategies for targeted ferroptosis therapy (Figure 3).

### 4.4 β-hydroxybutyrylation

Ferroptosis is a novel form of cell death driven by iron-dependent lipid peroxidation, and β-hydroxybutyrate (BHB) is one of the ketone bodies produced by the liver. Recent studies have demonstrated that the liver-specific ketone body, β-hydroxybutyrate (BHB), regulates ferroptosis by inducing histone β-hydroxybutyrylation modification in various disease models. The Zheng team was the first to elucidate BHB’s core protective mechanism in an acute liver failure (ALF) model: by enhancing H3K9bhb, it significantly reduced lipid peroxidation markers, such as malondialdehyde (MDA) and reactive oxygen species (ROS), thereby blocking the ferroptosis process (Zheng Y. et al., 2022). In exploring the indirect regulatory mechanisms between β-hydroxybutyrylation modification and ferroptosis, Yu et al.'s study revealed that in neurodegenerative disease models, such as Parkinson’s disease (PD), BHB inhibits lipid peroxidation and ferroptosis by upregulating ZFP36 (an RNA-binding protein) and suppressing ACSL4 protein expression (Yu X. et al., 2023). Tian’s results indicate that both exogenous and endogenous β-HB inhibit ferroptosis in kidneys by reducing ferroptosis markers (such as lipid peroxidation products like MDA and ACSL4) while upregulating anti-ferroptosis proteins (such as GPX4 and SLC7A11) (Tian et al., 2024). Interestingly, unlike lysine crotonylation (kla), lysine succinylation (ksucc), and other modifications that promote ferroptosis, H3K9bhb and substrates involved in histone β-hydroxybutyrylation, such as BHB and β-HB, appear to be protective factors. Their increased concentration can protect cells by reducing oxidative stress markers (like MDA) and upregulating GPX4 expression (Figure 3).

## 5 Ferroptosis-targeted molecule related to PTMs with potential for clinical translation

Ferroptosis is closely associated with the occurrence and progression of numerous diseases. However, the development of drugs targeting ferroptosis for therapeutic purposes faces significant challenges. Currently, there are no clinically approved drugs for the treatment of ferroptosis. The existing FSP1-specific inhibitor, iFSP1, is unsuitable for *in vivo* use because, at high concentrations, it exhibits off-target effects and fails to effectively target FSP1, thereby promoting cellular ferroptosis, which hinders its clinical translation. Therefore, it is crucial to identify new drug molecules with clinical translation potential for the treatment of ferroptosis (Table 5).

Research has revealed that novel post-translational modifications (PTMs) act as core molecular switches in the ferroptosis process, primarily by regulating lipid peroxide accumulation and disrupting redox homeostasis. Some compounds can directly target PTMs to modulate ferroptosis. For instance, Yu et al. found that evodiamine, a bioactive alkaloid, can inhibit H3K18la levels and subsequently increase Sema3A expression, which impairs angiogenesis. Furthermore, evodiamine induces ferroptosis by reducing the expression of glutathione peroxidase 4 (GPX4) (Yu Y. et al., 2023). Additionally, dexmedetomidine (Dex), an α_2_-adrenergic receptor agonist, alleviates myocardial injury by reducing the lactylation of malate dehydrogenase 2 (MDH2) (She et al., 2024).

In addition to the molecules mentioned above that influence ferroptosis through direct interaction with novel PTMs, some substances can affect ferroptosis through indirect regulatory mechanisms, particularly HDAC inhibitors and SIRT5-related ferroptosis regulation drugs.

Doxorubicin, an anti-tumor drug, has been reported to downregulate the expression of SLC7A11 and GPX4 in a dose-dependent manner at low concentrations (0.1 μM) as a highly selective inhibitor of HDAC1. Inhibition of the SLC7A11/GPX4 axis promotes lipid peroxidation in leukemia cells and further triggers ferroptosis (Yang G. et al., 2024). Moreover, Zhu et al. discovered Tectorigenin (TEC), a flavonoid monomer with hepatoprotective effects. Its tRNA-derived fragments (tRFs) bind to HDAC1 and regulate histone lactylation modifications at ferroptosis-driving genes ATF3, ATF4, and CHAC1, thereby inhibiting ferroptosis in hepatocytes (Zhu et al., 2025).

Trichostatin A (TSA), which inhibits HDACs, induces hyperacetylation of histones, activating the NRF2-KEAP1 signaling cascade and promoting the transcriptional upregulation of GPX4, thus inhibiting ferroptosis. Additionally, Oliveira et al. confirmed that HDAC inhibitors induce epithelial-to-mesenchymal transition (EMT) and upregulate transferrin receptor (TfR1) expression, causing the accumulation of free iron to threshold levels (52 μM) in SW13 cells, surpassing the ferroptosis initiation barrier (Oliveira et al., 2021). Similarly, in SIRT5 knockdown mice, liver pathology worsens, accompanied by elevated malondialdehyde (MDA) and iron levels, and increased expression of GPX4 (Li J. et al., 2024; Huang et al., 2024a). The mechanism involves the activation of the Nrf2/HO-1 signaling pathway through SIRT5 knockdown, which upregulates GPX4 expression, inhibiting ferroptosis and exacerbating ischemia-reperfusion injury in ischemic stroke. SIRT5 also enhances the binding of HOXA5 to the FSP1 promoter through its de-succinylation, upregulating ferroptosis inhibitor protein 1 (FSP1) expression, thereby inhibiting ferroptosis and alleviating septic lung injury (Wang et al., 2025b).

In addition to the aforementioned monotherapy, combination therapies can expand the application of existing inhibitors across various indications. Although the molecular mechanisms of ferroptosis regulation through novel PTMs are gradually becoming clearer, and targeted strategies have shown potential in preclinical models (such as in leukemia and solid tumors), there are currently no ferroptosis-targeted drugs based on HPTMs or HDAC inhibitors that have entered the clinical translation phase.

## 6 Summary and prospects

The study of novel post-translational modifications (PTMs) represents a broad and rapidly advancing field. Traditional modifications, including acetylation, methylation, and phosphorylation, are primarily enzyme-catalyzed, whereas novel modifications, such as lactylation and crotonylation, can be directly mediated by metabolites, thereby linking cellular metabolic states with epigenetic regulation.

By linking the regulation of cellular ferroptosis to disease progression, the potential for clinical translation has been further augmented. However, it is unfortunate that there is still limited research on novel PTM inhibitors targeting cellular ferroptosis sites. The development of new ferroptosis-targeted drugs based on PTMs or HDAC inhibitors still faces three major challenges: 1 Insufficient functional analysis of PTM modification sites. 2 The bidirectional regulatory mechanism of metabolic microenvironment fluctuations on the PTM-ferroptosis axis remains unclear. 3 Pharmacological strategies targeting PTM regulatory elements to intervene in ferroptosis need to be devised.

Recent studies demonstrate that NAA10-mediated lactylation of NSUN2 at residues K216 and K389 depletes glutathione levels and compromises GPX4 activity, thereby conferring resistance to ferroptosis in gastric cancer cells (An et al., 2024). This finding validates site-directed mutagenesis combined with functional phenotyping as a core strategy for determining PTM site functionality, establishing a transferable framework for investigating PTM sites in other ferroptosis-related proteins such as GPX4. In a distinct pathway, SIRT5 inhibits ferroptosis and ameliorates sepsis-induced lung injury by desuccinylating HOXA5 to upregulate FSP1 expression (Wang et al., 2025b). This observation raises an important question: can structural optimization yield specific SIRT5 inhibitors like MC3482 that mitigate undesirable off-target effects? Furthermore, on a positive note, in the field of epigenetics, a multitude of sophisticated technologies have been developed to investigate and elucidate the epigenomic status of the genome and its potential molecular mechanisms (Yao et al., 2024). Techniques such as NGS (Next-Generation Sequencing) (Chenarani et al., 2021), CUT&Tag (Li et al., 2021), and Third-Generation Sequencing (TGS) (Van Dijk et al., 2018) have provided new avenues for researching and exploring disease-related proteins and identifying epigenetic markers. In the future, further exploration of the spatiotemporal-specific interaction networks of different novel PTMs is essential. A deeper understanding of how these epigenetic mechanisms influence various diseases and the exploration of tissue-specific regulatory strategies will drive breakthroughs in epigenetics.
